# Guideline-Adherent Clinical Validation of a Comprehensive 170-Gene DNA/RNA Panel for Determination of Small Variants, Copy Number Variations, Splice Variants, and Fusions on a Next-Generation Sequencing Platform in the CLIA Setting

**DOI:** 10.3389/fgene.2021.503830

**Published:** 2021-05-20

**Authors:** Theresa A. Boyle, Ashis K. Mondal, Daryoush Saeed-Vafa, Sudha Ananth, Pankaj Ahluwalia, Ravi Kothapalli, Alka Chaubey, Evans Roberts, Dahui Qin, Anthony M. Magliocco, Amyn M. Rojiani, Ravindra Kolhe

**Affiliations:** ^1^Department of Pathology and Thoracic Oncology, H. Lee Moffitt Cancer Center and Research Institute, Tampa, FL, United States; ^2^Department of Pathology, Medical College of Georgia at Augusta University, Augusta, GA, United States

**Keywords:** next-generation sequencing, oncology, targeted panel, DNA variants, RNA variants, validation, clinical

## Abstract

We describe the clinical validation of a targeted DNA and RNA-based next-generation sequencing (NGS) assay at two clinical molecular diagnostic laboratories. This assay employs simultaneous DNA and RNA analysis of all coding exons to detect small variants (single-nucleotide variants, insertions, and deletions) in 148 genes, amplifications in 59 genes, and fusions and splice variants in 55 genes. During independent validations at two sites, 234 individual specimens were tested, including clinical formalin-fixed, paraffin-embedded (FFPE) tumor specimens, reference material, and cell lines. Samples were prepared using the Illumina TruSight Tumor 170 (TST170) kit, sequenced with Illumina sequencers, and the data were analyzed using the TST170 App. At both sites, TST170 had ≥98% success for ≥250× depth for ≥95% of covered positions. Variant calling was accurate and reproducible at allele frequencies ≥5%. Limit of detection studies determined that inputs of ≥50 ng of DNA (with ≥3.3 ng/μl) and ≥50 ng RNA (minimum of 7 copies/ng) were optimal for high analytical sensitivity. The TST170 assay results were highly concordant with prior results using different methods across all variant categories. Optimization of nucleic acid extraction and DNA shearing, and quality control following library preparation is recommended to maximize assay success rates. In summary, we describe the validation of comprehensive and simultaneous DNA and RNA-based NGS testing using TST170 at two clinical sites.

## Introduction

Historically, targeted sequencing of a limited group of clinically relevant genes has been most commonly practiced to optimize cost, turnaround times, and minimize reporting complexity (Beadling et al., 2013; Hadd et al., 2013; Singh et al., 2013; Luthra et al., 2014). However, this minimalistic approach can lead to an incomplete mutation profile and lacks comprehensive screening of all known hotspots and tumor-suppressor genes. This point is important because many heterogeneous tumors carry multiple mutations, and tumorigenesis can be altered by several types of variations, including single nucleotide variants (SNVs), small insertions or deletions (indels), amplifications, splice variations, and gene fusions. Furthermore, the use of different panels for DNA and RNA testing or for different tumor types within a laboratory results in inefficiencies in sample usage, time, training, and cost. Thus, there is value in larger gene panels that offer coverage of a broader set of clinically relevant genes for a wide range of tumor types, especially if they are compatible with lower nucleic acid input amounts.

Next-generation sequencing (NGS)-based approaches are fast becoming the standard of care for routine screening of clinical tumor samples. In a clinical laboratory, comprehensive oncology panels that can simultaneously evaluate multiple variant categories and be used for a broad range of tumor types hold significant appeal. However, the best approach for selecting, validating, and reporting results of a comprehensive NGS assay as a laboratory-developed test may not be readily apparent to all potential users.

In this study, we describe the clinical validation of an intermediate-sized 170-gene NGS pan-cancer assay, TruSight Tumor 170 (TST170), at two independent clinical molecular diagnostic laboratories. This panel was an extension of a smaller previously validated 26-gene NGS mutation panel, TruSight Tumor 26 (TST26), in these two laboratories which was optimized for use with formalin-fixed, paraffin-embedded (FFPE) tumor samples (Fisher et al., 2016). The TST170 panel allows for simultaneous analysis of DNA and RNA variants within 170 genes associated with solid tumors, including fusions and splice variants in 55 genes, single-nucleotide variants, insertions, and deletions in 148 genes, and amplifications in 59 genes, to detect tumor-specific information to guide diagnostic and molecular subclassification of tumors, biomarker-based prognostication, and variant-driven therapy choices. We realize that this validation of the TST170 assay may be just one more step forward in the path toward whole genome sequencing (WGS) and whole transcriptome sequencing (WTS) of cancer samples for patient care. Our practical experience in this implementation with adherence to the Association for Molecular Pathology (AMP)/College of American Pathologist (CAP) guidelines is presented and discussed to serve as a model for others considering the validation of this type of assay in their clinical laboratories.

## Materials and Methods

### Assay Selection and Guideline-Based Strategy

The choice of an NGS-based assay for use in a clinical setting requires careful consideration of the content of the panel, inclusion of evidence-based markers, and compatibility with patient specimen types that are typically received for testing. These patient samples are often limited in size and tumor cellularity. The comprehensive TST170 NGS-based panel described here incorporates protocols with integrated reagents, a workflow that facilitates analysis of most solid tumor-relevant DNA and RNA variants, and built-in bioinformatics that permit rapid laboratory implementation and reporting. The validation strategy for both laboratories was guided by the recently published AMP and CAP guidelines on validating NGS-based oncology panels and bioinformatics pipelines (Jennings et al., 2017; Roy et al., 2018) which included specific recommendations for test development, optimization, and validation (Jennings et al., 2017). For assay validation, they recommended the use of reference materials and cell lines to determine assay performance with the measurement of positive percent agreement and positive predictive value for each variant type, and provided rationale for the optimal number of clinical samples to use for evaluating performance.

### Samples Tested

Validation studies were performed independently at two clinical molecular diagnostic laboratories: Laboratory A, H. Lee Moffitt Cancer Center and Research Institute*;* Laboratory B, Georgia Esoteric and Molecular Laboratory at Augusta University. Methods are summarized in Supplementary Table 1. At both locations, AcroMetrix^TM^ Oncology Hotspot Control (Thermo Fisher Scientific, Fremont, CA) was used as a reference DNA control and SeraSeq Fusion RNA Mix v2 (SeraCare Life Sciences, Milford, MA) was used as a reference RNA control. The DNA control contained over 500 mutations from the Catalog of Somatic Mutations in Cancer (COSMIC) database across 53 genes, and included 5 variant types (SNVs, multiple nucleotide variants, deletions, insertions, and complex variants) of varying nucleotide lengths. The RNA control included 14 gene fusions, 1 exon-skipping variant, and 1 multi-exon deletion. At Laboratory A, cancer cell lines as part of the validation studies included: the GM24385 cell line engineered with the AcroMetrix^TM^ Control, Coriell Pool 1 with pooling of DNA from 10 different Coriell cell lines (Coriell), NCI-HD753 (Horizon Diagnostics), and NCI-596 (Horizon Diagnostics); no cell line material was used at Laboratory B beyond the GM24385 cell line AcroMetrix^TM^ Control. At both locations, FFPE patient specimens with known mutation profiles were tested during the validation studies. The patient specimens included a range of tumor types: lung, skin, CNS, breast, kidney, brain, head, and neck, GI and colon cancer, gall bladder cancer, soft tissue, bone, ovarian, endometrial, and other uterine cancers. Sample details are depicted in Supplementary Figure 1. Laboratory A evaluated a total of 54 patient specimens before clinical implementation by testing 10 different tumor types. Of these, 46 specimens were tested during primary validation to assess assay performance and 8 additional specimens were evaluated for pathologist training and standardization of reporting prior to clinical implementation. Results of these eight specimens were reviewed by all six pathologists assigned to report TST170 results. Laboratory B evaluated a total of 173 patient specimens covering more than 15 different tumor types. For both laboratories, tumor cellularity of the specimens ranged from 10 to 100%. A minimum tumor cellularity of 10% was required prior to macro-dissection to enrich tumor cellularity and inclusion in clinical accuracy evaluations.

### Nucleic Acid Extraction

To prepare for nucleic acid extraction, hematoxylin and eosin-stained tissue sections were reviewed by a molecular pathologist to evaluate tumor content and annotate the tumor area. After evaluation, 2–5 unstained 5 μM tissue sections were deparaffinized and tumor tissue was manually macro-dissected. At Laboratory A, DNA and RNA were extracted simultaneously using the AllPrep DNA/RNA FFPE Kit (QIAGEN, United States). Laboratory A performed a later validation of 7-μM thick sections which showed concordance with results from 5-μM thick sections. At Laboratory B, DNA was extracted using the QiAMP DNA FFPE Tissue Kit (QIAGEN; FFPE surgical or cell preparation blocks) or the DNeasy Tissue Kit (QIAGEN; FNA or cytology cell preparations), and RNA from FFPE surgical or cell preparation blocks was extracted using the miRNeasy FFPE Kit (P/N- 217504, QIAGEN, United States). RNA from fine needle aspirations (FNAs) or cytology cell preparations were extracted using the RNeasy Mini Kit (P/N-74104, QIAGEN, United States). Manufacturer recommendations were followed for the quantitative and qualitative evaluation of DNA and RNA quality. Nucleic acids were quantitated using Qubit (Invitrogen, Carlsbad, CA). At Laboratory A, DNA and RNA quality was evaluated by running DNA and RNA Screen Tape in Tape Station (Agilent Technologies 2200 or 4200) to ensure samples had a DV-200 ≥20%. At Laboratory B, DNA quality was assessed by a NanoDrop spectrophotometer, and OD 260/280 values between 1.7 and 2.2 were considered acceptable. At Laboratory B, RNA quality was assessed using NanoDrop and dsRNA was measured by Qubit with the HS RNA kit. Samples that met quality thresholds were diluted with RNase/DNase free water, as required, to adjust the nucleic acid concentration to the desired value. The TST170 assay protocol was optimized for a defined DNA input range of 40–120 ng total (at a concentration of 3.3—10 ng/μl) and RNA input range of 40–85 ng total (4.7—10 ng/μl).

### Library Preparation

Library preparation was performed using the hybrid capture-based TST170 Library Preparation Kit (Illumina, San Diego, CA) following the manufacturer’s protocol (TST170 Reference Guide) (Illumina, 2019). Briefly, DNA was fragmented using a Covaris Focused-Ultrasonicator (Covaris, Woburn, MA) to generate DNA fragments of 90–250 base pairs (bp), with a target peak of approximately 130 bp. Total RNA from each sample was denatured and primed with random hexamers and then double-stranded cDNA was synthesized and cleaned up. Both gDNA and cDNA libraries were prepared simultaneously with the same methods and workflow. Samples next underwent end repair, A-tailing, and adapters were added to the ends of gDNA and cDNA by ligation. Both adapter-ligated gDNA and cDNA fragments were amplified by index PCR using DNA and RNA-specific index primers (using unique index primer mixes and combinatorial index primer mixes) to add index sequences for sample multiplexing. At this stage, the nucleic acid fragments were flanked by the sequences and adaptors required for cluster generation. The samples underwent two rounds of hybridization and capture to bind targeted regions of the DNA and RNA and optimally enrich the libraries. The enriched libraries underwent PCR amplification, cleanup with sample purification beads, and quantification by Qubit. Bead-based normalization of the enriched libraries was performed to ensure a uniform library representation in the pooled libraries before sequencing.

### Sequencing and Data Analysis

**FIGURE 1:**
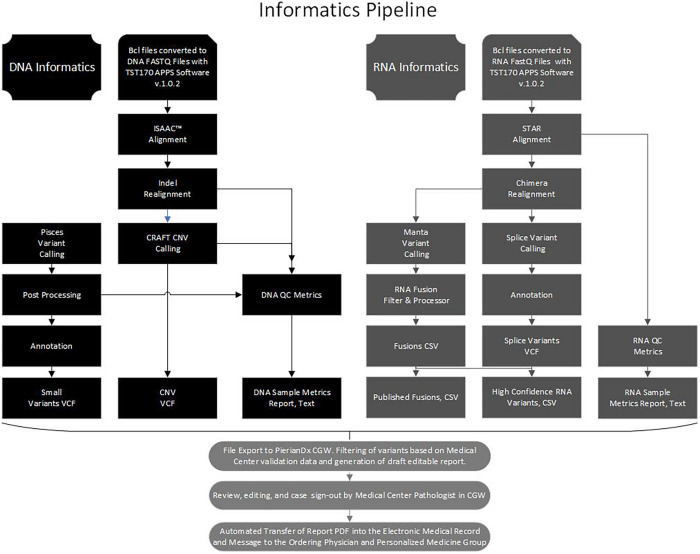
Bioinformatics pipeline. Flow chart to illustrate the processing of the DNA and RNA sequence data with software in the Illumina Basespace Apps to generate five DNA and RNA VCF, CSV, and TXT files for export to the PierianDx Clinical Genomics Workstation (CGW) for filtering and reporting.

Libraries were sequenced on a NextSeq 500 or 550 (Illumina), with 16 libraries (8 DNA and 8 RNA) sequenced per run to achieve maximum sample coverage. The sequence BCL files were processed through the BaseSpace TST170 App (Illumina) with generation of DNA and RNA fastQ files. The fastQ files were further processed with several software programs into five files, a small variants variant call file (VCF), a copy number variants (CNV) VCF, a DNA sample quality metrics text (TXT) file, a high confidence RNA variants file (CSV), a published fusion file (CSV), and a RNA sample metrics (TXT) file (Figure 1). At Laboratory A, the fastQ, BAM, and the files processed by the TST170 App were subsequently transferred, filtered, and reported with Clinical Genomics Workspace (CGW) software from Washington University using a PierianDx informatics pipeline. These files were filtered and annotated to generate a preliminary draft report of the detected variants in a tiered manner based on clinical significance. Variants were called based on genomic build GRCh37.p13 and genomic annotation sources NCBI RefSeq v105. Databases used for variant filtering and reporting included dbSNP (149), ClinVar (20170905), COSMIC (v80), ExAC (v1.0), dbNSFP (3.0b2c), and NHLBI ESP (v.0.0.30) (Supplementary Table 2). DNA and cDNA sequences were viewed with Integrated Genomics Viewer (IGV, a publicly available visualization tool generously offered by the Broad Institute).

At Laboratory B, results from the App were subsequently transferred and reported using multiple resources, including IBM Watson for Genomics (IBM WfG) and the above described CGW software from Washington University. During the validation process, both laboratories also evaluated alternative reporting software (e.g., SophiaGenetics, IBM Watson). Each identified substitution, indel, CNV, splice site variant, and fusion was reported according to the joint consensus recommendations of the AMP, American Society of Clinical Oncology (ASCO), and CAP in the “Standards and Guidelines for the Reporting of Sequence Variants in Cancer” (Li et al., 2017).

Two filters were tested separately for substitutions and indels: filter one was set for a read depth ≥100× and variant allele frequency ≥2.6%, and filter two was set for a read depth ≥250× and variant allele frequency ≥5%. Standard filters were set as recommended by the manufacturer: low variant quality (GQ ≥ 20), low depth (DP ≥ 100), strand bias (SB ≤ 0.5 or 3.01 converted to GATK convention), low variant frequency (VF ≥ 0.026), indel reference repeat (homopolymer stretch ≤8), adjusted quality (AQ ≥ 10), and low variant support (≥7).

The filter for CNVs was based on the identification of the CNVs in the TST170 BaseSpace App. The filter for fusions and splice site variants was based on the “high confidence” filter in the TST170 BaseSpace App which uses a Manta-based algorithm. A second filter for “low confidence” fusions was established using an alternative fusion algorithm developed at PierianDx.

### Precision and Reproducibility Studies

The DNA AcroMetrix^TM^ Control and RNA SeraSeq v2 control specimens were repeated within runs to assess reproducibility, between runs to assess repeatability, with two different operators, and with two different sequencers to assess robustness of variant detection. Results were analyzed separately for substitutions, indels, CNVs, and fusions/splice variants.

### Analytical Sensitivity (Limit of Detection) Studies

Both laboratories evaluated substitution and indel variants across a wide range of known variant allele frequencies by diluting the DNA AcroMetrix^TM^ Control with wild-type DNA from a patient specimen. At Laboratory A, the DNA AcroMetrix^TM^ Control was diluted for the evaluation of four concentrations (100, 50, 33, and 25%). Later, additional validation studies of lower concentrations were performed with dilutions of Coriell Pool 1–4, 3, 2, and 1 ng/μl and five clinical samples with dilutions to 4, 2, and 1 ng/μl. At Laboratory B, 50 ng of the DNA AcroMetrix^TM^ Control was diluted for the evaluation of eight concentrations (100, 84, 68, 50, 33, 17, 8.5, and 0%). Similarly, dilution of the RNA SeraSeq v2 control with wild-type RNA from a patient specimen allowed the evaluation of the ability to detect translocations and splice variants at lower concentrations. Laboratory A evaluated RNA at four concentrations (100, 50, 33, and 25%). At Laboratory B, 64 ng of the RNA control was diluted to evaluate eight different concentrations (100, 75, 50, 25, 5, 1, 0.5, and 0%).

### Analytical Specificity Studies

At both laboratories, assay specificity was determined by testing specimens with potentially interfering substances, such as a lung cancer specimen with anthracosis (black pigment in lung caused by pollutants such as smoke), a melanoma specimen with melanin, a bone specimen with decalcification, a specimen with a history of freezing in OCT compound for frozen section diagnosis, and a pleural effusion.

### Accuracy Studies

Cell lines and synthetic materials were used to test a wide range of clinically important substitutions, indels, and fusions/splices site variants. Patient specimens were used for accuracy testing of CNVs and to confirm the clinical diagnostic sensitivity and specificity of each variant category.

At Laboratory A, 44 patient samples and 2 additional control specimens (CAP, Northfield, IL) were used to evaluate accuracy. All samples were previously characterized using one or more of the following methods: DNA pyrosequencing, NGS by FoundationOne or Guardant360, NGS by Oncocomplete, immunohistochemistry for mismatch repair protein loss, microsatellite instability testing, and/or fluorescence in-situ hybridization (FISH). Calculations for control specimens were performed separately from clinical specimens.

At Laboratory B, reference and clinical materials were used to allow for the testing of a wide range of clinically important substitutions, indels, and fusions/splices site variants. Patient specimens were used for analysis of accuracy of all the variants and to confirm clinical diagnostic sensitivity. Diagnostic specificity of all variants was determined using routinely processed specimens available at the Augusta University. Samples included 173 patient samples, 5 known CAP proficiency specimens [KRAS-01 (Catalog number 30586843), BRAF-01 (Catalog number 29705306), and EGFR-01 (Catalog number 29705894)], and patient samples previously characterized by real time PCR (Roche), Asuragen’s QuantideX^®^ NGS Cancer Hotspot 21, and NGS by Foundation One.

For the DNA control evaluations, accuracy was evaluated over a range of coverage and sequence contexts. Substitutions and indels called from replicate tests of the Acrometrix^TM^ DNA control were compared to publicly available data for this control provided by the manufacturer and the Personal Genome Project whole genome sequence for the background cell line (GM24385). Coding positions of all genes covered by the DNA component of the TST170 assay were assessed using the assay target bed file to define the target space.

For RNA control evaluations, accuracy was assessed over a range of fusion copy numbers. Fusion and splice variant calls from replicate tests of the SeraSeq fusion RNA mix v2 in its undiluted form were compared to manufacturer data. Only fusions and splice site variants that passed the high confidence filter were analyzed. A fusion was identified as detected if the breakpoint fell within the expected exons reported by SeraCare.

For SNVs, a result was counted as a true positive (TP) if a variant at that position was identified in the validation study and was annotated as a variant in the gold standard. A false positive (FP) result was defined as a variant identified by the validation study which was not identified in the gold standard. A false negative (FN) result was defined as a variant called in the gold standard but not in the validation study, despite achieving sufficient coverage (data analyzed using ≥100× or ≥250× as different cut-offs to define sufficient coverage). Finally, the remaining positions within analyzed regions with sufficient coverage (≥100× or ≥250×) but no variants identified were counted as true negative (TN) calls. If an FN position achieved a coverage depth less than the defined coverage (100× or 250×) in the validation dataset, it was referred to as a “No Call.” For fusions and splice variants, a TP was defined as a fusion call that passed the Illumina high confidence filter; FN calls could be due to either the fusion not being called or the fusion being called but filtered out (low confidence fusion).

Accuracy was determined by comparing the positive percentage agreement [PPA = TP/(TP+FN)], negative percentage agreement [NPA = TN/(TN+FP)], positive predictive value [PPV = TP/(TP+FP)], and negative predictive value [NPV = TN/(TN+FN)] for each variant type (substitutions, indels, CNVs, and fusions/splice variants).

## Results

### Assay Demonstrated Precise and Reproducible Results

The results summarized in Table 1 show the average PPA determined in reproducibility experiments. Evaluated variants spanned a range of allele frequencies and copy numbers for a realistic assessment of reproducibility. Of the different variant types, CNVs performed best with 100% agreement for all comparisons. Substitutions were detected with greater than 95% concordance in all categories at both laboratories. Indels demonstrated lower concordance (>75%) in all categories at Laboratory A, which likely reflects that indels are inherently more difficult to detect and that some of the indels had variant allele frequencies at or below the TST170 limit of detection. Concordance for fusions/splice variants was over 80% for all categories; the lower concordance observed at Laboratory A was mainly driven by lower concordance of three specific fusions, *TMPRSS2-ERG*, *SLC34A2-ROS1*, and *EGFR-SEPT14.* Inconsistent detection of *TMPRSS2-ERG* and *SLC34A2-ROS1* fusions was likely lower due to these two fusions having lower copies in the control than most of the other fusions. *TMPRSS2-ERG* had only six copies/ng in the control, which is below the seven copies/ng lower limit of detection claimed for the TST170 assay by Illumina. Detection of the *EGFR-SEPT14* fusion was likely compromised by a repetitive sequence near the fusion junction. Incidentally, Laboratory A continued to use the same DNA and RNA controls after going live with the assay and *EGFR-SEPT14* fusion was more frequently detected, possibly due to the increased laboratory experience with the assay.

**TABLE 1:** Analytical precision.

**Variant type (n)**	**N**	**Read depth; filter**	**Intra-run PPA (%)**	**Inter-run PPA (%)**	**Inter-operator PPA (%)**	**Inter-machine PPA (%)**
**Laboratory A**						
Substitutions	790	≥100×; VAF ≥2.6%	96.6	96.3	96.5	97.5
Indels	110	≥250×; VAF ≥5%	82.7	78.8	76.2	84.5
CNVs	59	“pass”	100	100	100	100
Fusions and splice variants	15	“high confidence”	84.3	81.4	93.8	92.6
Number pairwise comparisons		NA	3	3	1	8
**Laboratory B**						
Substitutions	876	≥250×; VAF ≥5%	>99	>99	>99	ND
Indels	102	≥250×; VAF ≥5%	>99	>99	>99	ND
CNVs	51	“pass”	100	100	100	ND
Fusions and splice variants	42	“high confidence”	>99	>99	>99	ND
Number pairwise comparisons		NA	3	5	3	ND

*CNV, copy number variant; NA, not applicable; ND, not determined; PPA, positive predictive agreement; VAF, variant allele frequency.*

### Analytical Sensitivity Drops Sharply With Inputs Lower Than 50 ng

Hotspot analysis of the diluted DNA specimens revealed a decrease in assay sensitivity with sample dilution, as expected. Experiments at Laboratory A revealed poor sensitivity for substitutions (71%) and indels (62%) detection at the 1:1 dilution. These results suggest that substitutions and indels cannot be reliably detected with inputs lower than the 40 ng recommended by the manufacturer. Larger input amounts of ≥50 ng provided more reliable detection. Similarly, analysis of diluted RNA also showed poor sensitivity (64%) at the 1:1 dilution suggesting that fusions/splice variants may not be reliably detected with input amounts below 40 ng or 7 copies/ng. Analytical specificity experiments revealed detection of expected genetic alterations in all specimens, including specimens from frozen section blocks, mild decalcification, high melanin, and anthracosis (cigarette pollutant in lungs) with no detected effect of the tested substances on assay performance.

After the initial validation at Laboratory A, and due to clinical demand for testing of specimens with lower than ideal DNA concentrations, Laboratory A performed additional dilution studies to further characterize sensitivity of variant detection at very low DNA concentrations. At times, specimens with low concentrations are the only specimen available for testing and there might be delays, health risks and financial loss associated with gaining another specimen, if even possible. With the standard Laboratory A protocol, 50 μl of extracted DNA diluted to 4 ng/l was subjected to fragmentation, but only 25 μl of the fragmented DNA (equivalent to 100 ng) was used for downstream sequencing. Therefore, up to 25 μl of additional fragmented DNA volume beyond the 25 μl used per the standard protocol was available for sequencing studies of samples with lower concentrations. For example, with a 1 ng/μl concentration, instead of using only 25 μl of fragmented DNA, the entire volume of 50 μl could be used for a total input of 50 ng. With this protocol alteration, even though the concentration of 1 ng/μl was below the Illumina recommended range of 3.3–10 ng/μl, the ultimate input of 50 ng was within the Illumina recommended range of 40–120 ng total input.

Sequencing of Coriell Pool 1 (DNA pooled from 10 different cell lines) was performed with dilutions of 4, 3, 2, and 1 ng/μl. Sequencing of five previously reported clinical specimens was performed with dilutions of 2 and 1 ng/μl (routine sequencing previously performed with standard 4 ng/μl dilutions). Variants detected in Coriell Pool 1 by TST170 at 4, 3, and 2 ng/μl were 100% concordant with each other and with prior results from sequencing with the TST26 gene panel (33 variants). One difference though was that two intronic variants were filtered out in the TST170 informatics pipeline that had not been filtered out in the prior TST26 informatics pipeline. At 1 ng/μl, 31 of 33 variants were detected but 2 variants were not detected in the *CDH1* and *CTNNB1* genes. As expected, sample quality control metrics, such as total reads, on target reads, and average coverage, decreased with lower concentrations and most metrics for samples with concentrations of 1 ng/μl were below the minimum reference range limit. In the 5 patient specimens, 40 variants deemed clinically significant or potentially significant in the original reported results were identified in the samples with 2 ng/μl concentration; 37 of the 40 variants were detected in the 1 ng/μl samples. Qualified testing of 43 patient specimens with DNA concentrations of >2 and <3.3 ng/μl produced results with adequate sequence metrics for reporting as qualified reports and results appeared concordant with results of other genetic sequencing, such as liquid biopsy results from the testing of circulating tumor DNA. They were reported with clear communication with the oncologists and with a qualifying statement regarding the higher chance for false negative results.

**FIGURE 2:**
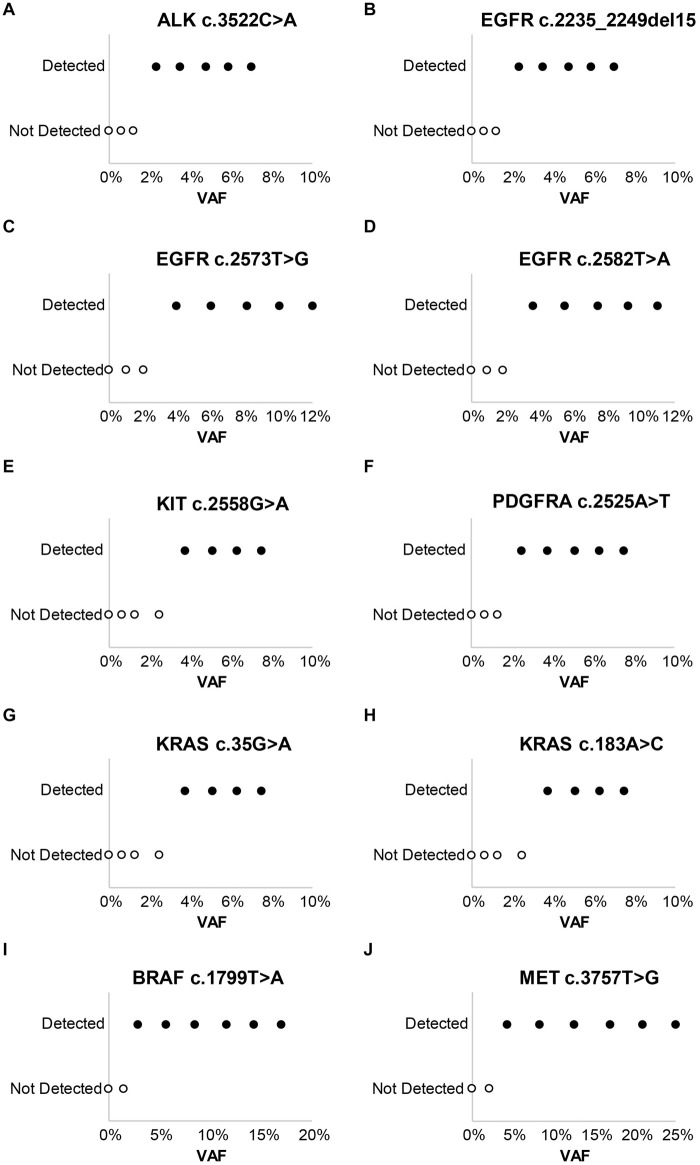
Evaluation of assay limit of detection for DNA variants. Progressive dilution of the DNA control, and subsequent reduction in the variant allele frequency (VAF), results in a reduction in assay sensitivity for substitutions and indels. **(A)** *ALK* c.3522C > A. **(B)** *EGFR* c.2235_2249del15. **(C)** *EGFR* c.2573T > G. **(D)** *EGFR* c.2582T > A. **(E)** *KIT* c.2558G > A. **(F)** *PDGFRA* c.2525A > T. **(G)** *KRAS* c.35G > A. **(H)** *KRAS* c.183A > C. **(I)** *BRAF* c.1799T > A. **(J)** *MET* c.3757T > G.

**FIGURE 3:**
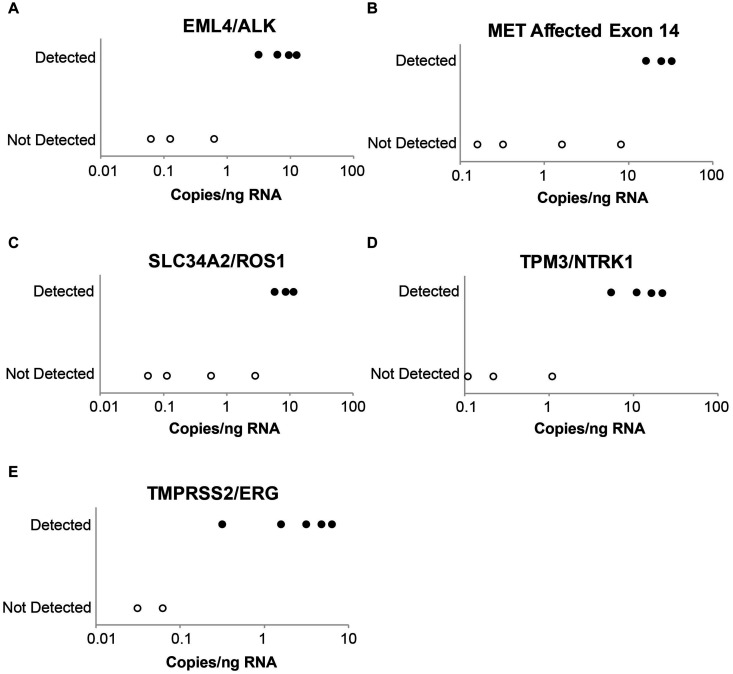
Evaluation of assay limit of detection for RNA variants. Progressive dilution of the RNA control results in a reduction in assay sensitivity for fusions and splice variants. **(A)** *EML4*-*ALK*. **(B)** *MET* affected exon 14. **(C)** *SLC34A2*-*ROS1*. **(D)** *TPM3*-*NTRK1*. **(E)** *TMPRSS2*-*ERG*.

Laboratory B similarly evaluated the influence of DNA and RNA dilution on the detection of known variants. Representative plots for a selection of clinically relevant SNVs in *ALK*, *EGFR*, *KIT*, *PDGFRA*, *KRAS*, *BRAF*, and *MET* are depicted in Figure 2. The two variants with the highest allele frequencies (>15%; *BRAF* c.1799T > A and *MET* c.3757T > G) in the DNA control sample were detected down to the second lowest dilution containing DNA (17%). Variants were not detected in the negative control (0% DNA) sample. Dilution of the RNA control sample eventually resulted in a loss of fusion and splice variant detection; the limit of detection varied between different RNA variants (representative examples are depicted in Figure 3).

The capacity of the assay to detect mutations at low tumor cellularity was evaluated at Laboratory A, with variant detection rates determined for samples with a tumor cellularity as low as 10%. Substitutions, indels, and fusions/splice site variants were detected as expected with tumor cellularity as low as 10%.

### Assay Is Accurate for Small Variants in Controls at Low Allele Frequency

Assay accuracy was determined using control specimens as shown in Table 2. Based on AMP and CAP guidelines, Laboratories A and B used the same controls but used the control data differently during the validation analysis. It was analyzed as separate data in Laboratory A and combined data in Laboratory B. Table 2 reflects the differences in how the control data were used differently in the validation analyses. Both laboratories A and B selected variant allele frequency ≥5% and read depth ≥ 250× as filters with the minimum thresholds for making a positive call for substitutions with consideration for the need to balance high sensitivity and low FP call rate. Laboratory A also analyzed the data with a filter of ≥3% and read depth of 100× which had higher sensitivity and, in control specimens, a retained low FP rate. Laboratory A decided for clinical samples to use the higher threshold of ≥5% for automatic placement of substitutions on the report, and to put variants in the 3–5% range in a “variants for review” tab to allow the sign-out pathologist an opportunity to view the sequence for these variants in IGV and decide whether or not to add them to the report on a case by case basis, dependent on clinical scenario, diagnosis, sequence quality, and presence of the variant in both the DNA and cDNA sequence.

**TABLE 2:** Accuracy analysis.

**Variant type**	**Read depth; filter**	**TP**	**TN**	**FP**	**FN**	**PPA (%)**	**NPA (%)**	**PPV (%)**	**NPV (%)**
**Laboratory A, control specimens**									
Substitutions	≥100×; VAF ≥2.6%	10652	7349439	303	73	99.3	99.9	97.2	99.9
Indels	≥250×; VAF ≥5%	594	7359678	180	75	88.8	99.9	76.7	99.9
Fusions/splice variants	“high confidence”	301		15	67	81.8		95.2	
**Laboratory A, clinical specimens**									
Substitutions	≥100×; VAF ≥2.6%	41^∧^			0	100			
Indels	≥250×; VAF ≥5%	5^†^			0	100			
Copy number variants		14			2	87.5			
Fusions/splice variants		17^‡^			1^§^	94.4			
**Laboratory B, combined data for control and clinical specimens**									
Substitutions	≥250×; VAF ≥5%					99.87	100	100	98.33
Indels	≥250×; VAF ≥5%					97.56	100	100	97.43
Copy number variants	Filter pass, > 7 copies					96.87	100	100	97.67
Fusions/splice variants	“high confidence”					97.87	100	100	98.36

*^∧^Includes 3 substitutions with low variant support or low variant frequency.*

*^†^Includes one indel with low adjusted quality score.*

*^‡^Nine fusions were detected as high confidence fusions, two fusions were detected as low confidence fusions.*

*^§^The fusion not detected (ETV1-DGKB) was reported as a not previously characterized fusion in the FoundationOne report. VAF, variant allele frequency; TP, true positive; TN, true negative; FP, false positive; FN, false negative; PPA, positive percentage agreement; NPA, negative percentage agreement; PPV, positive predictive value; NPV, negative predictive value.*

During variant review in IGV, the cDNA sequence was viewed in the top and the DNA sequence in the bottom with both sequences was aligned to the Genome Reference Consortium Build 37 (GRCh37.p13). The ability to view both the DNA and cDNA sequence on the same screen in IGV can be very helpful for the confirmation of variants with lower allele frequency in the DNA sequence. This single screen view orthogonal verification is particularly helpful with small biopsies or low tumor cellularity resection samples when a mutation may be difficult to confidently call as a true positive with only the DNA sequence. For example, if a BRAF p.V600E mutation is present at 3% allele frequency in the DNA sequence of a low tumor cellularity melanoma sample, if it is also present in the cDNA sequence, it may be reported with more confidence. For indels, both Laboratories A and B selected a filter with a variant allele frequency ≥5% and a read depth of ≥ 250× to balance high sensitivity and a low FP rate.

### RNA Fusion Controls Provide Helpful Information

Evaluation of fusion and splice variant results at Laboratory A using Manta fusion calling software revealed that 54 of the 67 (81%) FN results were caused by repeated missed detection of 3 of the 16 fusions/splice variants in the SeraCare reference material (SeraSeq fusion RNA mix v2). Two of the fusions, *SLC34A2*-*ROS1* and *TMPRSS2*-*ERG*, could not be detected reliably, likely because of an inherent lower number of copies of these fusions in the control than most of the other fusions, with *TMPRSS2-ERG* having a particularly low concentration of six copies/ng, below the limit of detection (LOD) of seven copies/ng claimed by the technical specifications for the TST170 assay. Another fusion variant in the reference material, *EGFR*-*SEPT14*, had a highly repetitive sequence at the splice junction. In discussion with the manufacturer (Illumina) and secondary reporting informatics companies (PierianDx and IBM WfG), it was determined that a highly repetitive sequence at the splice junction was complicating detection. In retrospect, one option that might have been helpful for troubleshooting in this situation would have been to analyze the RNA FastQ files through a different fusion calling software program, such as Delly or Trans-ABySS. With inclusion of the three fusions, the PPA was 81.8% which was reported in the validation; however, exclusion of the three fusions would increase the PPA to 95.5%.

At Laboratory B, the accuracy of fusion and splice site variant detection using this assay was evaluated using the same SeraCare RNA control. The RNA control was run in undiluted form on multiple runs and results were compared to available data on the SeraCare website. Only fusions and splice site variants that passed the TST170 App high confidence filter were analyzed. A fusion was called positive if the breakpoint fell within the expected exons reported by SeraCare. A TP result in this analysis was defined as a fusion call that passed the Illumina high confidence filter. FN calls could be due to either the fusion not being identified or the fusion being called but filtered out (low confidence fusion). One fusion variant, *EGFR*-*SEPT14*, consistently not reported by the commercial reporting tools (PierianDx and IBM WfG), was detected by the TST170 app but filtered out later. Laboratory B decided to include the failure to detect *EGFR*-*SEPT14* fusions in the validation data since the laboratory was planning use of PierianDx and IBM WfG for clinical result reporting. Inclusion of this fusion in the analysis resulted in a PPA of 97%, NPA of 100%, PPV of 100%, and NPV of 95%.

### Assay Is Accurate for Small Variants, Amplifications, Fusions, and Splice Variants With Clinical Specimens

A total of 219 clinical specimens across the two sites were evaluated with results shown in Table 2. At Laboratory A, the variants within the clinical specimens encompassed 41 known substitutions from prior testing. All 41 substitutions were concordantly detected by the TST170 assay, although three had low variant support or low variant frequency. Nine specimens had prior pyrosequencing results with the identification of clinically important mutations in the *EGFR*, *KRAS*, *BRAF*, and *NRAS* genes. Five specimens had indels detected by prior sequencing with TST26. All were detected by TST170, although one indel detected in *MSH6* had a low adjusted quality score. The longest indel detected was a 15 bp deletion in *EGFR* exon 19. The PPA for CNVs with a minimum call threshold of 4 copies was 87.5% with comparison to 16 variants detected on FoundationOne NGS reports. Any CNV different from the expected number of two copies was included in the analysis. A total of 12 fusions and 6 splice site variants were present in the evaluated clinical samples. Nine fusions [*EML4-ALK*, *KIF5B*-*RET (x2)*, *TMPRSS2*-*ERG (x2)*, *EPS15*-*NTRK1*, *FGFR2*-*TACC2*, *NOTCH2*-*SEC22B*, and *GOPC*-*ROS1*] were detected as high confidence fusions. Two fusions were detected as low confidence fusions with an alternative pipeline that puts detected fusions into a “low confidence” tab (*TMPRSS2*-*ERG* exhibited low duplicate reads, *IGH-BCL2* was reported as imprecise/low quality). One novel fusion, *ETV1*-*DGKB*, that has not been characterized in the literature was expected per the FoundationOne report but was not detected during the validation run, even though the *ETV1* gene was covered by the panel. The sample tested was from a patient with diffuse large B cell lymphoma and the FoundationOne report showed a high tumor mutation burden of 46 mutations per megabase (Mb). All splice site variants, including *MET* exon 14 skipping, *EGFR*vIII, and *BCL2*, were identified as expected. With the inclusion of only high confidence fusions, the PPA was 83.3%. With the inclusion of low confidence fusions, the PPA increased to 94.4%. Based on these data, a decision was made to have low confidence fusions in a tab for review of the sequence by the sign-out pathologist in the context of the clinical scenario.

At Laboratory B, 24 variants were analyzed from testing of the 173 clinical specimens (see Table 2: *NRAS* p.Q61K, *BRAF* p.V600K, *BRAF* p.V600E, *BRAF* p.D594N, *IDH1* p.R132H, *PTEN* p.R130Q, *EGFR* p.T790M, *EGFR* p.S678I, *EGFR* p.G719X, *GNAS* p.R201C, *GNAS* p.R201H, *KRAS* p.G13D, *KRAS* p.G12A, *KRAS* p.G12D, *KRAS* p.G12C, *KRAS* p.G12V, *KRAS* p.A146V, *FBXW7* p.R441C, *TP53* p.G154C, *TP53* p.P142L, *TP53* p.G266E, *DNMT3A* p.R882C, *KIT* p.V559D, *KIT* p.V654A). All these variants were detected as expected. For indels, a variety of clinical specimens were included in the validation analysis. These clinical specimens represented multiple in-frame insertions, frameshift variants, and in-frame deletions, which were all detected. Of these, the majority were DNA specimens with prior pyrosequencing results that represented clinically important mutations, such as *EGFR*, *KRAS*, *BRAF*, and *NRAS*. The remaining samples had NGS sequencing results from the Asuragen QuantideX^®^ NGS Cancer Hotspot 21 panel or FoundationOne. Five specimens had indels with the longest being a 15 bp deletion in *EGFR* exon 19 and all five were detected. The clinical specimens included 12 fusions and 6 splice site variants. All the fusions were detected as high confidence fusions [*SRPK2-BRAF*, *GTF2I-BRAF*, *CLIP2-BRAF*, *ALK-EML4* (2 cases), *EWSR1-ATF1*, *EWSR1-WT1*, *ANKRD29-AR*, *JDP2-AR*, *RET-IKBK*, *EWSR1-FLI1* (5 independent samples), *PVT1*-*MYC*, and *GOPC-ROS1*]. All splice site variants, including *MET* exon 14 skipping and *EGFR*vIII, were identified as expected. There were 36 clinical samples with 12 copy number variants detected by FISH, SNP microarray (Oncoscan), and/or NGS (Foundation One); all variants with a copy number above 7 were detected. There were multiple variants (8) in clinical samples detected by SNP microarray with a copy number below 4, and these were not detected by the panel. Use of a high confidence filter of ≥7 copy numbers resulted in a PPA of 96.87%, NPA of 100%, PPV of 100%, and NPV of 97.67%.

### Assay Has Excellent Coverage of Over 3,000 Transcripts

**FIGURE 4:**
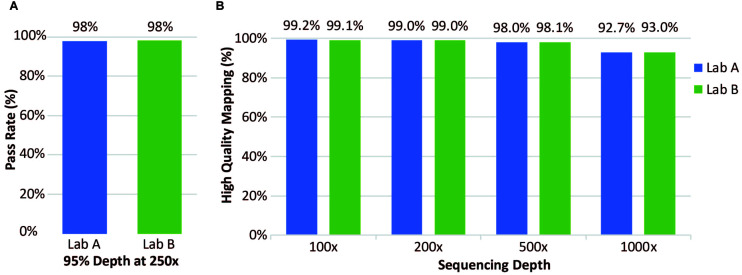
Coverage of clinical samples. **(A)** Coverage pass rate, where coverage was considered to pass when the depth was at least 95% at 250×. **(B)** Average percentage of high-quality mapped reads at four different sequencing depths.

Clinical samples were also analyzed for coverage across thousands of transcripts. At both laboratories, coverage was considered to “pass” when depth of reads was ≥250× in ≥95% of positions. Laboratory A evaluated 21 clinical specimens; Laboratory B evaluated 72 clinical specimens; both laboratories had a pass rate of 98% (Figure 4A). Laboratory A reported 65 of the 3064 transcripts with one or more failure for at least one specimen. Exclusion of one low quality specimen reduced the failure count from 65 to 50 transcripts. This analysis highlighted the effect of lower specimen quality on coverage and identified 50 transcripts with lower coverage. Mapping analysis revealed that 96% of reads were mapped and 98% had high-quality unique on-target mapping. The average percentage with high-quality mapping decreased with increasing depth but remained above 92% up to 1,000× depth (Figure 4, right).

At Laboratory B, 72 clinical specimens were analyzed for coverage. The pass rate for coverage was 98% with coverage defined as a “pass” for all exons when depth of reads was ≥250× in ≥95% of positions, same as Laboratory A (Figure 4A). Only a small percentage of transcripts analyzed had one or more failure for at least one specimen (Figure 4B). Overall, this assay performed well with excellent coverage of the included genes.

### Implementation Considerations: Turn Around Time and Reporting

One very important consideration with clinical NGS testing is turnaround time (TAT). The TST170 workflow from nucleic acid extraction to variant calling can be completed in around 4 days, which includes around 32 h for library preparation and 24 (NextSeq) to 27 (HiSeq 2500) h of sequencing. The TATs for both Laboratory A and B for routine testing are less than 2 weeks from receipt in their respective laboratories; for time-sensitive cases, a faster TAT of approximately 1 week has been achieved. During the validation process the authors evaluated several bioinformatics pipelines in addition to the locked TST170 App from the manufacturer, running the BAM files through different analysis platforms (e.g., PierianDx, IBM Watson for Genomics, Sophia Genetics, Philips IntelliSpace, and QIAGEN Clinical Insight (QCI). These comparisons were helpful for identifying strengths and weaknesses of the platforms used. An ideal variant reporting system would include a combined tier and level approach with adherence to the tier system for variant reporting following CAP/ACMG/AMP guidelines (Li et al., 2017) and level-based reporting that aligns with NCCN guidelines. The final implementation and reporting of the validated TST170 assay at the two laboratories was similar, with both laboratories following professional reporting recommendations (Li et al., 2017; New York State Department of Health, 2018).

At Laboratory A, the validated TST170 assay, termed the Moffitt Solid Tumor Actionable Results (Moffitt STAR^TM^) NGS panel, was implemented as the primary assay for evaluating solid tumors, replacing the TruSight Tumor 26 (TST26) 26-gene assay used previously. With the announcement of its launch for solid tumor testing in February 2018, it was acknowledged that TST170 does not cover all variants of clinical value for neurology and sarcoma solid tumors and that testing of these solid tumors may require use of other assays. The final bioinformatics pipeline used for Moffitt STAR^TM^ includes processing fastQ files in the TST170 App in Illumina BaseSpace Enterprise for alignment, variant calling, and initial filtering to generate small variant, CNV, high confidence RNA variants, and DNA and RNA sample metric files for output (Roberts et al., 2017). These files along with the fastQ and BAM files are transferred to the PierianDx CGW and further filtered and annotated to generate a preliminary draft report of detected variants in a tiered manner based on clinical significance. DNA and cDNA sequences are viewed by the pathologist with IGV. The draft reports are manually reviewed and edited by the sign-out molecular pathologist such that genetic alterations with higher clinical significance appear at the top of the report with concise manual interpretations, variants of potential significance appear next with usually more brief interpretations and, below this, the variants of uncertain significance are listed in a table. The final PDF report is automatically transferred into the electronic medical record upon sign-out.

At Laboratory B, solid tumor samples were previously evaluated using an independent genetics laboratory. After validation of the TST170 assay, the assay was named Augusta OncoTarget^TM^ and genetic testing migrated from send-out to in-house testing; two staggered library preparations and sequencing runs are performed each week. For sarcomas, a fusion panel is used as an add-on to the Augusta OncoTarget^TM^ assay. Laboratory B viewed the implementation of in-house testing as desirable because of the reduction in time to reporting.

For both laboratories, a standard cut-off of 5% variant allele frequency is employed for reporting substitutions, insertions, and deletions, and a “high confidence” pass filter is used for reporting fusions. For amplifications, Laboratory A uses a cut-off of 4 copies to report and Laboratory B uses a cut-off of 7 copies and ≤1 copy for larger deletions. For splice variants, the sequence for splice variants called by the Illumina splice variant “high confidence” filter is reviewed to determine whether the sequence characteristics are adequate for reporting. In general, reporting requires at least 20 sequence reads to support the presence of a splice variant. For both laboratories, if small variants have a VAF between 3 and 5%, the sample tumor cellularity and the sequence quality with IGV software is reviewed by the pathologist and, if deemed an accurate call, the variant is reported. For cases with VAF lower than 5%, the ability to simultaneously evaluate cDNA sequence in addition to DNA sequence for regions with both DNA and RNA coverage (as designed to occur for the most clinically actionable regions) can be a significant advantage. Furthermore, the RNA component of TST170 has the capacity to detect rare fusions with just one of the two genes involved in the fusion covered on the panel. This allows for the detection of rare and novel fusions. For example, an expected *EML4-ALK* fusion and an unexpected *AGK-BRAF* fusion was detected in a lung cancer sample harboring a known *EML4-ALK* fusion at Laboratory A during the TST170 validation and was confirmed by orthogonal testing with a different NGS assay (Boyle et al., 2020).

Post-implementation evaluation of prevalence of CNVs, fusions, and splice variants is ongoing to compare TST170 NGS results with other results of testing of the same patients to gain a better understanding of the clinical utility of these results for this assay. In addition, synthetic CNV reference material from SeraCare, such as SeraCare^®^ Breast Cancer CNV mix + 3 copies (5 total copies), became available after implementation and has subsequently been incorporated into the quality control samples included with the clinical runs at both laboratories. The *ERBB2*, *FGFR3*, and *MYC* genes each had low level amplification as expected (5 copies for *ERBB2* and *FGFR3* and 4 copies for *MYC*). Currently, if the CNV result is considered potentially actionable (e.g., might inform treatment selection), orthogonal testing with a more traditional CNV assay such as immunohistochemistry or RNA *in situ* hybridization for *ERBB2* (HER2) is recommended to confirm results.

## Discussion

### Comprehensive Genomic Profiling Has Advantages and Challenges

Historically, clinical laboratories have utilized small gene panels, often targeted to a specific tumor type. Small panels offer some benefits, such as relatively simple interpretation, but can have major drawbacks. Specifically, small gene panels may not include all variants required for identification of appropriate therapeutics and/or clinical trials, prognosis, and diagnosis of different tumor types/subtypes. Sequential use of small panels can lead to sample exhaustion. In contrast, larger gene panels allow for maximal use of precious/limited samples and provide comprehensive analysis of tumor variants (Frampton et al., 2013). The TST170 assay targets all coding exons of 170 genes, per the current RefSeq database (O’Leary et al., 2016), with the genes and variant types selected based on evidence-based recommendations from professional organizations, such as the (European Society for Medical Oncology, 2021; National Comprehensive Cancer Network, 2021), independent consortia publications, and late-stage pharmaceutical research. Although TST170 provides comprehensive coverage of variants in genes likely to play a role in the tumorigenesis of solid tumors, offering it for clinical care is just one step on the way toward WGS and whole transcriptome sequencing (WTS) for patient care. Illumina is now also offering a 500 gene panel, TSO500, that has been validated for clinical use in several clinical laboratories around the world. This sharing of the practical NGS validation experience of two academic laboratories with the TST170 assay described here is intended as a general example of how to perform a guideline adherent NGS validation with limited resources. The strategies used might also be applicable to validation of other NGS assays, including TSO500.

With more comprehensive genetic profiling, assay analysis and interpretation are more complex. One specific limitation with the TST170 assay, and NGS-based panels in general, is that detection of CNVs is less developed and more limited than FISH-based assays. Unlike FISH where there is a centromere marker, NGS does not have a simple marker for chromosome number. As such, with NGS, it can be difficult to differentiate between true gene amplification and amplification due to changes in chromosome number or structure. Although not the focus of this report, this assay can be used to evaluate TMB and MSI (Zhang et al., 2017a, b). TMB and MSI reporting was not validated by either Laboratory A or B during the initial TST170 validation. However, in light of recent changes to colon cancer guidelines with a recommendation for MSI testing in all colon cancer patients (National Comprehensive Cancer Network, 2019), and the FDA approval of MSI as a biomarker for targeted therapy with immunotherapy, both laboratories later performed additional validations for MSI reporting, Laboratory A with TST170 and Laboratory B with Illumina TSO500. TMB reporting was also validated by Laboratory B with TSO500.

### The TST170 Assay Is Robust and Accurate Even With Small Samples

In this study, a total of 219 clinical samples across the two laboratories in addition to cell lines and control samples were used to determine assay LOD and validate TST170 performance. Although each laboratory followed their own validation protocols, there were general similarities in the validation experiments and outcomes. The TST170 assay is compatible with Illumina NextSeq 500/550 and HiSeq 2500 sequencers, allowing up to 16 libraries (8 DNA/8 RNA) to be analyzed per run with Illumina NextSeq 500/550 and 12 libraries (6 DNA, 6 RNA) on HiSeq 2500 sequencers, making this assay suitable for mid- to high-volume routine testing in a clinical molecular laboratory. Here, both laboratories ran the TST170 assay on NextSeq sequencers. Evaluation of DNA and RNA controls demonstrated good reproducibility across operators, runs, and sites for each variant type. LOD experiments suggested that using DNA or RNA input amounts lower than those recommended by the manufacturer (40 ng) would result in a reduction in analytical sensitivity for all variant types. For routine clinical samples, both laboratories set the minimum standard pre-macro-dissection tumor cellularity at 10%, although even with this low cut-off, pathologist discretion is permitted. The tumor quantity, cellularity, and quality are considered at the time of result interpretation with description of the higher risk for false negative results for specimens with lower quantity/quality, as needed. Evaluation of cell lines and clinical samples with previous testing results revealed high diagnostic sensitivities and concordance across variant types at both laboratories.

### Key Methods and Lessons Learnt

Prior to commencing validation experiments, both laboratories performed initial testing to establish standard operating procedures and best practices. Although the general assay workflow was the same at the two laboratories, there were some differences. For example, Laboratory A performed co-extraction of DNA and RNA while Laboratory B performed separate DNA and RNA extractions. The authors determined that optimization of several protocol steps was crucial to maximize assay performance and minimize assay failure. Comparison of mechanical shearing approaches identified mechanical shearing, ideally with a Covaris instrument as recommended by the assay manufacturer (Illumina Inc, 2019), as a critical step and required purchase of a new instrument, Covaris ME220, by Laboratory A and specific setting up of a Pulsing Protocol. Both laboratories targeted a mean DNA fragment size of 130 bp. The other key step identified was quality control of library preparation before sequencing. Laboratory B determined that employing a minimum nucleic acid input of 50–120 ng (DNA) and 50–85 ng (RNA) virtually eliminated test failures (data not shown), and now use these values as the minimum input requirement for clinical samples. For samples where RNA library preparation fails (e.g., insufficient RNA), but the DNA library preparation is satisfactory, DNA results are reported out with a statement regarding the lack of RNA results. Some recommended best practices from the laboratories include incorporating STOP and fatigue points in the run sheet; tracking of lot numbers for each sample/run; and recording of the user and date performed for each step of the protocol. Both laboratories implemented the cloud-based option, BaseSpace Enterprise, for security and storage reasons, with data remaining identified throughout processing. A local server-based version of BaseSpace is also available. While the server-based approach may offer some cost benefits, the additional work required in using this option was viewed as undesirable by both laboratories.

### Organization of the SEQUencing for Oncology Informatics Academic (SEQUOIA) Team

During validation, open discussion and collaboration between laboratories dealing with the same validation and pipeline challenges was needed. An informal face-to-face meeting of colleagues from five laboratories leading TST170 assay validations at their sites was arranged in conjunction with the 2017 AMP meeting. The informal group at this meeting self-organized as the SEQUencing for Oncology Informatics Academic (SEQUOIA) team. The SEQUOIA Team has grown into an international consortium of over 30 independent laboratories. Teleconferences are scheduled once a month to share questions, ideas, troubleshoot results, quality assurance tips, and progress. Strengths and weaknesses of the assay and pipelines, along with solutions, are discussed freely in a manufacturer-independent environment. Ongoing SEQUOIA projects include tumor mutation burden (TMB) and microsatellite instability (MSI) validation and standardization of reporting.

### Final Thoughts

In summary, using a wide range of tumor and sample types, we have shown that the TST170 assay can detect somatic mutations present in FFPE solid tumor samples at 5% mutant allele frequency with greater than 95% sensitivity and specificity. With consideration of the clinical scenario and specialized review of DNA and cDNA sequence quality in IGV, variants with lower allele frequencies may be reported with confidence. The ability to visualize both DNA and cDNA sequence in IGV allows for accurate identification of many potentially targetable genetic changes, including point mutations, insertions, deletions, CNVs, fusions, and splice variants. Many alterations detected by this panel, such as *BRAF* and *EGFR* mutations and *ALK, ROS1, RET, NTRK1, NTRK2*, and *NTRK3* fusions, have potential as companion diagnostics for FDA-approved therapies. Detection of other alterations can help with clinical decisions by providing diagnostic, prognostic, and therapeutic information. In this era of precision medicine, many therapies are in clinical trial pipelines and TST170 testing can help identify potential patients with clinical trials with biomarker inclusion criteria. Validation of the TST170 assay with its full DNA exon coverage of most genes, coverage of important RNA targets, and a high sensitivity with often small clinical FFPE samples is one step further along the path of interrogating tumors for actionable targets for patient care. With decreases in genetic sequencing costs and increases in actionable targets, profiling of the entire genome and transcriptome for patient care may be possible in the future. This assay identifies alterations with known targeted therapies, is helpful for selecting patients for clinical trials and later line therapies, and has the potential to be used as a companion diagnostic test for targeted therapeutics.

## Data Availability Statement

The data analyzed in this study is subject to the following licenses/restrictions: The datasets for this manuscript are not publicly available because of HIPAA regulations. Requests to access the datasets should be directed to TB, theresa.boyle@moffitt.org.

## Author Contributions

TB, RKot, DQ, AMM, and RKol contributed to the conception and design of the study. TB and RKol wrote the first draft of the manuscript. TB, RKol, DS-V, SA, PA, RKot, AC, ER, and AR compiled data, contributed to figures design, and to sections of the manuscript. All authors contributed to manuscript revision, read and approved the submitted version.

## Conflict of Interest

RK has received honoraria, travel funding, and research support from Illumina, Asuragen, QIAGEN, and BMS. TB has received honoraria and travel funding from Illumina and BMS. Library preparation kits and technical writing assistance were provided by Illumina. The remaining authors declare that the research was conducted in the absence of any commercial or financial relationships that could be construed as a potential conflict of interest.

## References

[B1] Beadling C. Neff T. L. Heinrich M. C. Rhodes K. Thornton M. Leamon J. (2013). Combining highly multiplexed PCR with semiconductor-based sequencing for rapid cancer genotyping. *J. Mol. Diagn.* 15 171–176. 10.1016/j.jmoldx.2012.09.003 23274167

[B2] Boyle T. A. Quinn G. P. Schabath M. B. Munoz-Antonia T. Saller J. J. Duarte L. F. (2020). A community-based lung cancer rapid tissue donation protocol provides high-quality drug-resistant specimens for proteogenomic analyses. *Cancer Med.* 9 225–237. 10.1002/cam4.2670 31747139 PMC6943158

[B3] European Society for Medical Oncology (2021). Available online at: http://www.esmo.org/ (accessed April 8, 2021).

[B4] Fisher K. E. Zhang L. Wang J. Smith G. H. Newman S. Schneider T. M. (2016). Clinical validation and implementation of a targeted next-generation sequencing assay to detect somatic variants in non-small cell lung, melanoma, and gastrointestinal malignancies. *J. Mol. Diagn.* 18 299–315. 10.1016/j.jmoldx.2015.11.006 26801070 PMC4816706

[B5] Frampton G. M. Fichtenholtz A. Otto G. A. Wang K. Downing S. R. He J. (2013). Development and validation of a clinical cancer genomic profiling test based on massively parallel DNA sequencing. *Nat. Biotechnol.* 31 1023–1031. 24142049 10.1038/nbt.2696 PMC5710001

[B6] Hadd A. G. Houghton J. Choudhary A. Sah S. Chen L. Marko A. C. (2013). Targeted, high-depth, next-generation sequencing of cancer genes in formalin-fixed, paraffin-embedded and fine-needle aspiration tumor specimens. *J. Mol. Diagn.* 15 234–247. 10.1016/j.jmoldx.2012.11.006 23321017

[B7] Illumina I. (2019). *TruSight Tumor 170 Reference Guide [Online].* Available online at: https://support.illumina.com/downloads/trusight-tumor-170-reference-guide-1000000024091.html (accessed April 8, 2021).

[B8] Illumina Inc (2019). *TruSight Tumor 170 Consumables & Equipment [Online].* Available: https://support.illumina.com/sequencing/sequencing_kits/trusight-tumor-170-kit/documentation.html (accessed April 8, 2021).

[B9] Jennings L. J. Arcila M. E. Corless C. Kamel-Reid S. Lubin I. M. Pfeifer J. (2017). Guidelines for validation of next-generation sequencing-based oncology panels: a joint consensus recommendation of the association for molecular pathology and college of american pathologists. *J. Mol. Diagn.* 19 341–365. 10.1016/j.jmoldx.2017.01.011 28341590 PMC6941185

[B10] Li M. M. Datto M. Duncavage E. J. Kulkarni S. Lindeman N. I. Roy S. (2017). Standards and guidelines for the interpretation and reporting of sequence variants in cancer: a joint consensus recommendation of the association for molecular pathology, american society of clinical oncology, and college of american pathologists. *J. Mol. Diagn.* 19 4–23. 27993330 10.1016/j.jmoldx.2016.10.002 PMC5707196

[B11] Luthra R. Patel K. P. Reddy N. G. Haghshenas V. Routbort M. J. Harmon M. A. (2014). Next-generation sequencing-based multigene mutational screening for acute myeloid leukemia using MiSeq: applicability for diagnostics and disease monitoring. *Haematologica* 99 465–473. 10.3324/haematol.2013.093765 24142997 PMC3943309

[B12] National Comprehensive Cancer Network (2019). *NCCN Clinical Practice Guidelines in Oncology: Colon Cancer v2.2019 [Online].* Available online at: https://www.nccn.org/professionals/physician_gls/pdf/colon.pdf (accessed June 7, 2019 2019).

[B13] National Comprehensive Cancer Network (2021). Available online at: https://www.nccn.org/ (accessed April 8 2021).

[B14] New York State Department of Health (2018). *Oncology Molecular Checklist: Next Generation Sequencing (NGS) Guidelines for Somatic Genetic Variant Detection [Online].* Available online at: https://www.wadsworth.org/sites/default/files/WebDoc/3NextGenSeqONCOGuidelines%2012318.pdf (accessed May 31, 2018).

[B15] O’Leary N. A. Wright M. W. Brister J. R. Ciufo S. Haddad D. Mcveigh R. (2016). Reference sequence (RefSeq) database at NCBI: current status, taxonomic expansion, and functional annotation. *Nucleic Acids Res.* 44 D733–D745. 26553804 10.1093/nar/gkv1189 PMC4702849

[B16] Roberts E. Boyle T. Magliocco A. Qin D. Moscinski L. Kothapalli R. (2017). “Validation of the moffitt solid tumor actionable result (STAR) NGS assay using the TST170 Panel by Illumina,” in *Proceeding of the28th International Molecular Med TRI-CON*, (San Francisco, CA).

[B17] Roy S. Coldren C. Karunamurthy A. Kip N. S. Klee E. W. Lincoln S. E. (2018). Standards and guidelines for validating next-generation sequencing bioinformatics pipelines: a joint recommendation of the association for molecular pathology and the college of american pathologists. *J. Mol. Diagn.* 20 4–27. 10.1016/j.jmoldx.2017.11.003 29154853

[B18] Singh R. R. Patel K. P. Routbort M. J. Reddy N. G. Barkoh B. A. Handal B. (2013). Clinical validation of a next-generation sequencing screen for mutational hotspots in 46 cancer-related genes. *J. Mol. Diagn.* 15 607–622. 10.1016/j.jmoldx.2013.05.003 23810757

[B19] Zhang S. So A. Kaplan S. Kruglyak K. (2017a). “Comprehensive evaluation of illumina’s trusight^®^ tumor 170 panel to estimate tumor mutational burden,” in *Proceeding of the AACR Annual Meeting*, (Washington, D.C).

[B20] Zhang S. So A. S. Kaplan S. Yao J. Kruglyak K. (2017b). “Assessment of microsatellite instability with Illumina TruSight^®^ Tumor 170 Panel,” in *Proceeding of the AMP 2017 Annual Meeting*, (Salt Lake, UT).

