# Supplementary material for: Guideline-Adherent Clinical Validation of a Comprehensive 170-Gene DNA/RNA Panel for Determination of Small Variants, Copy Number Variations, Splice Variants, and Fusions on a Next-Generation Sequencing Platform in the CLIA Setting
Source: Front Genet. 2021 May 20;12:503830. doi: 10.3389/fgene.2021.503830 (PMC8172991; doi:10.3389/fgene.2021.503830)
Supplement: Supplementary file 1 [file Table_1.DOCX]

**Supplementary Table 1**. Summary of methods used at each laboratory.

| **Laboratory Instrument or Method** | **Laboratory A, Moffitt** | **Laboratory B, Augusta University** |
| --- | --- | --- |
| RNA extraction | AllPrep DNA/RNA FFPE Kit (Qiagen, Cat #80234) | miRNAEasy FFPE Kit (Qiagen, Cat # 217504) |
| DNA extraction |  | QiAMP DNA FFPE Tissue Kit (Qiagen, Cat # 56404) |
| DNA/RNA Quantification | DNA quantity: Qubit dsDNA BR Kit (Q32850)  RNA quantity: Qubit RNA BR Assay Kit (ThermoFisher, Part #Q10210); Qubit Fluorometer (ThermoFisher)  RNA quality: Agilent Tapestation (Agilent Technologies 2100 Bioanalyzer®) | DNA quantity: Qubit dsDNA BR Kit (Q32850)  DNA quality: Nanodrop spectrophotometer |
| Library Prep | TruSight Tumor 170 (Illumina) | TruSight® Tumor 170 (Illumina) |
| DNA Shearing | Covaris (ME220 Focused-ultrasonicator™); target 130 bp fragment size | Covaris (ME220 Focused-ultrasonicator™); target 130 bp fragment size |
| Sequencer | NextSeq® 500 (Illumina) | NextSeq® 550 (Illumina) |
| Analysis | BaseSpace Enterprise, TST170 App® (Illumina) | BaseSpace Enterprise, TST170 App® (Illumina) |
| Secondary Analysis & Reporting | Clinical Genomicist Workstation (PierianDx)  SOPHIA DDM (SOPHiA™ GENETICS)  Variant Studio (Illumina)  IBM Watson for Genomics (IBM) | Clinical Genomicist Workstation (PierianDx)  Variant Interpreter (Illumina)  IBM Watson for Genomics (IBM) |

Cat #, catalogue number.
