# Supplementary material for: Guideline-Adherent Clinical Validation of a Comprehensive 170-Gene DNA/RNA Panel for Determination of Small Variants, Copy Number Variations, Splice Variants, and Fusions on a Next-Generation Sequencing Platform in the CLIA Setting
Source: Front Genet. 2021 May 20;12:503830. doi: 10.3389/fgene.2021.503830 (PMC8172991; doi:10.3389/fgene.2021.503830)
Supplement: Supplementary file 2 [file Table_2.DOCX]

**Supplementary Table 2**. Database details used for variant calling, filtering, and annotation during the validation

| Genomic Build: GRCh37.p13  Genomic Annotation Sources: NCBI RefSeq v105  dbSNP: 149  ClinVar: 20170905  COSMIC: v80  ExAC: v1.0  dbNSFP: 3.0b2c  NHLBI ESP: v.0.0.30 |
| --- |
