# Supplementary material for: Guideline-Adherent Clinical Validation of a Comprehensive 170-Gene DNA/RNA Panel for Determination of Small Variants, Copy Number Variations, Splice Variants, and Fusions on a Next-Generation Sequencing Platform in the CLIA Setting
Source: Front Genet. 2021 May 20;12:503830. doi: 10.3389/fgene.2021.503830 (PMC8172991; doi:10.3389/fgene.2021.503830)
Supplement: Supplementary file 3 [file Data_Sheet_1.PDF]

Supplementary Figure 1. Depiction of variants detected in control and clinical samples evaluated during the validation experiments at laboratory A (A) and laboratory B (B), with clinical samples sorted by tumor type. There are 5 genes listed that are not on the TST170 assay, which represent the unknown fusion partner for fusions detected by TST170 in clinical samples. The first three columns in each plot depicts whether the assay is designed to detect a specific variant type for a given gene, with a black square indicating coverage of that variant type in the indicated gene. For the next two columns for the DNA and RNA controls employed at the laboratories, a black square indicates that the manufacturer has indicated there is a variant present in that gene. Subsequent columns indicate whether an SNV (green), indel (red), amplification (yellow), or fusion/splice variant (blue) was detected in the sample when analyzed by TST170; at laboratory A, 3 additional color codings were used to indicate samples with multiple variants detected in a given gene, amplification and fusion/splice variant (lighter blue), amplification and SNV (orange), and amplification and fusion/splice variant and SNV (purple); at laboratory B, each variant within a gene for a specific sample was indicated in a separate column.

A

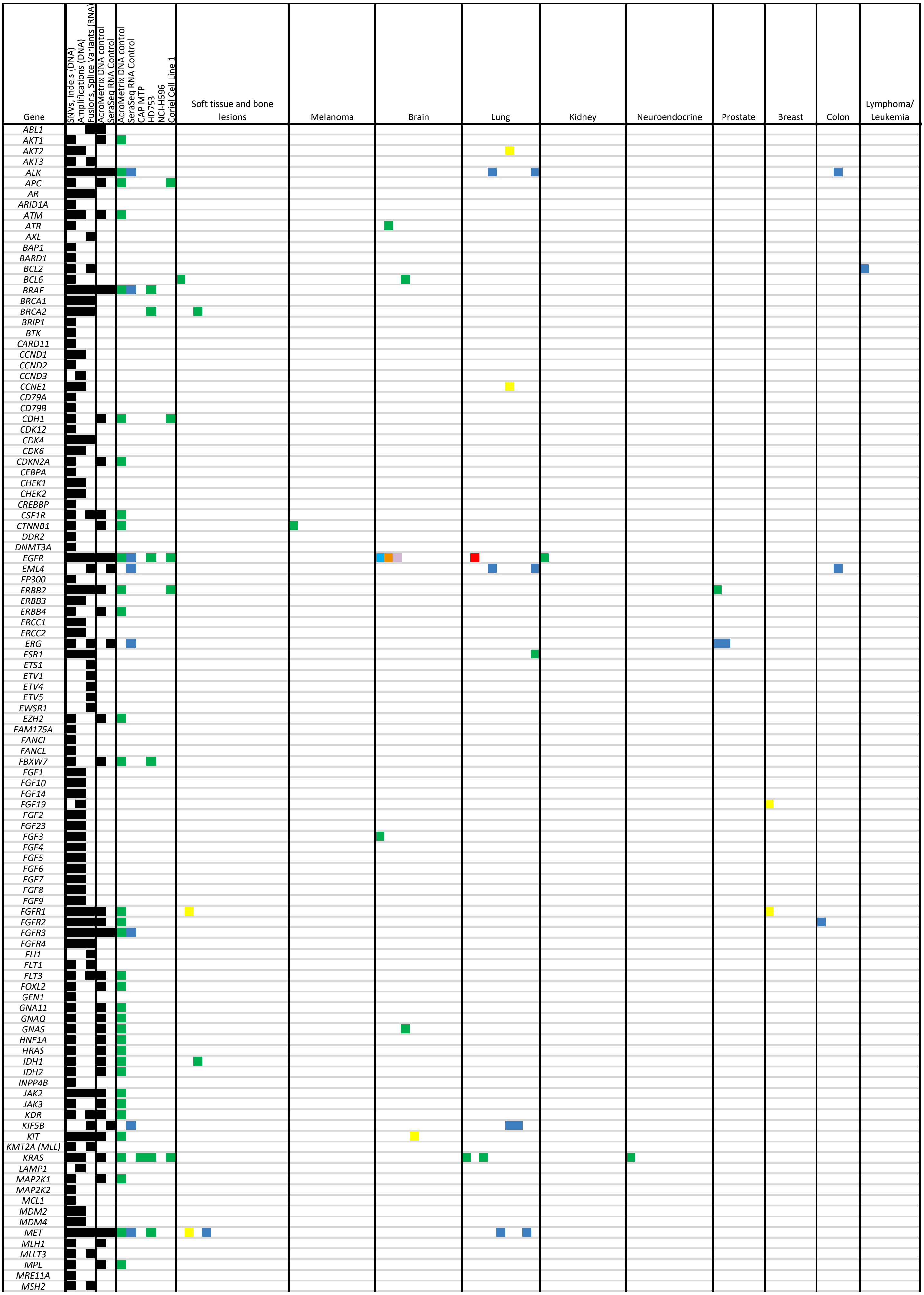
Gene
SNVs, Indels (DNA)
Amplifications (DNA)
Fusions, Splice Variants (RNA)
AcroMetrix DNA control
SeraSeq RNA Control
AcroMetrix DNA control
SeraSeq RNA Control
CAP MTP
HD753
NCI-H596
Coriel Cell Line 1
Soft tissue and bone lesions
Melanoma
Brain
Lung
Kidney
Neuroendocrine
Prostate
Breast
Colon
Lymphoma/ Leukemia
ABL1
AKT1
AKT2
AKT3
ALK
APC
AR
ARID1A
ATM
ATR
AXL
BAP1
BARD1
BCL2
BCL6
BRAF
BRCA1
BRCA2
BRIP1
BTK
CARD11
CCND1
CCND2
CCND3
CCNE1
CD79A
CD79B
CDH1
CDK12
CDK4
CDK6
CDKN2A
CEBPA
CHEK1
CHEK2
CREBBP
CSF1R
CTNNB1
DDR2
DNMT3A
EGFR
EML4
EP300
ERBB2
ERBB3
ERBB4
ERCC1
ERCC2
ERG
ESR1
ETS1
ETV1
ETV4
ETV5
EWSR1
EZH2
FAM175A
FANCI
FANCL
FBXW7
FGF1
FGF10
FGF14
FGF19
FGF2
FGF23
FGF3
FGF4
FGF5
FGF6
FGF7
FGF8
FGF9
FGFR1
FGFR2
FGFR3
FGFR4
FLI1
FLT1
FLT3
FOXL2
GEN1
GNA11
GNAQ
GNAS
HNF1A
HRAS
IDH1
IDH2
INPP4B
JAK2
JAK3
KDR
KIF5B
KIT
KMT2A (MLL)
KRAS
LAMP1
MAP2K1
MAP2K2
MCL1
MDM2
MDM4
MET
MLH1
MLLT3
MPL
MRE11A
MSH2

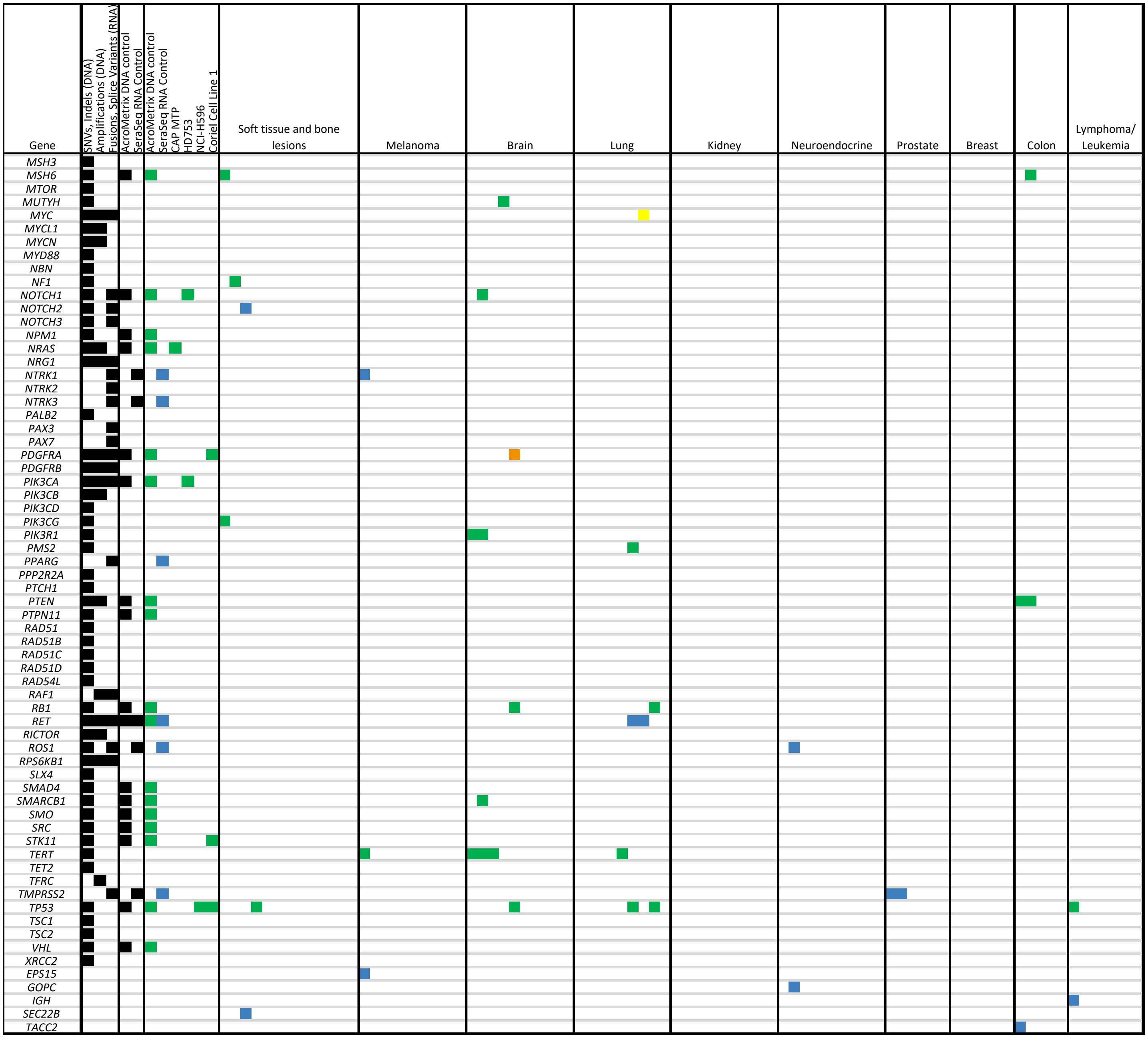
Gene
SNVs, Indels (DNA)
Amplifications (DNA)
Fusions, Splice Variants (RNA)
AcroMetrix DNA control
SeraSeq RNA Control
AcroMetrix DNA control
SeraSeq RNA Control
CAP MTP
HD753
NCI-H596
Coriel Cell Line 1
Soft tissue and bone lesions
Melanoma
Brain
Lung
Kidney
Neuroendocrine
Prostate
Breast
Colon
Lymphoma/ Leukemia
MSH3
MSH6
MTOR
MUTYH
MYC
MYCL1
MYCN
MYD88
NBN
NF1
NOTCH1
NOTCH2
NOTCH3
NPM1
NRAS
NRG1
NTRK1
NTRK2
NTRK3
PALB2
PAX3
PAX7
PDGFRA
PDGFRB
PIK3CA
PIK3CB
PIK3CD
PIK3CG
PIK3R1
PMS2
PPARG
PPP2R2A
PTCH1
PTEN
PTPN11
RAD51
RAD51B
RAD51C
RAD51D
RAD54L
RAF1
RB1
RET
RICTOR
ROS1
RPS6KB1
SLX4
SMAD4
SMARCB1
SMO
SRC
STK11
TERT
TET2
TFRC
TMPRSS2
TP53
TSC1
TSC2
VHL
XRCC2
EPS15
GOPC
IGH
SEC22B
TACC2

B

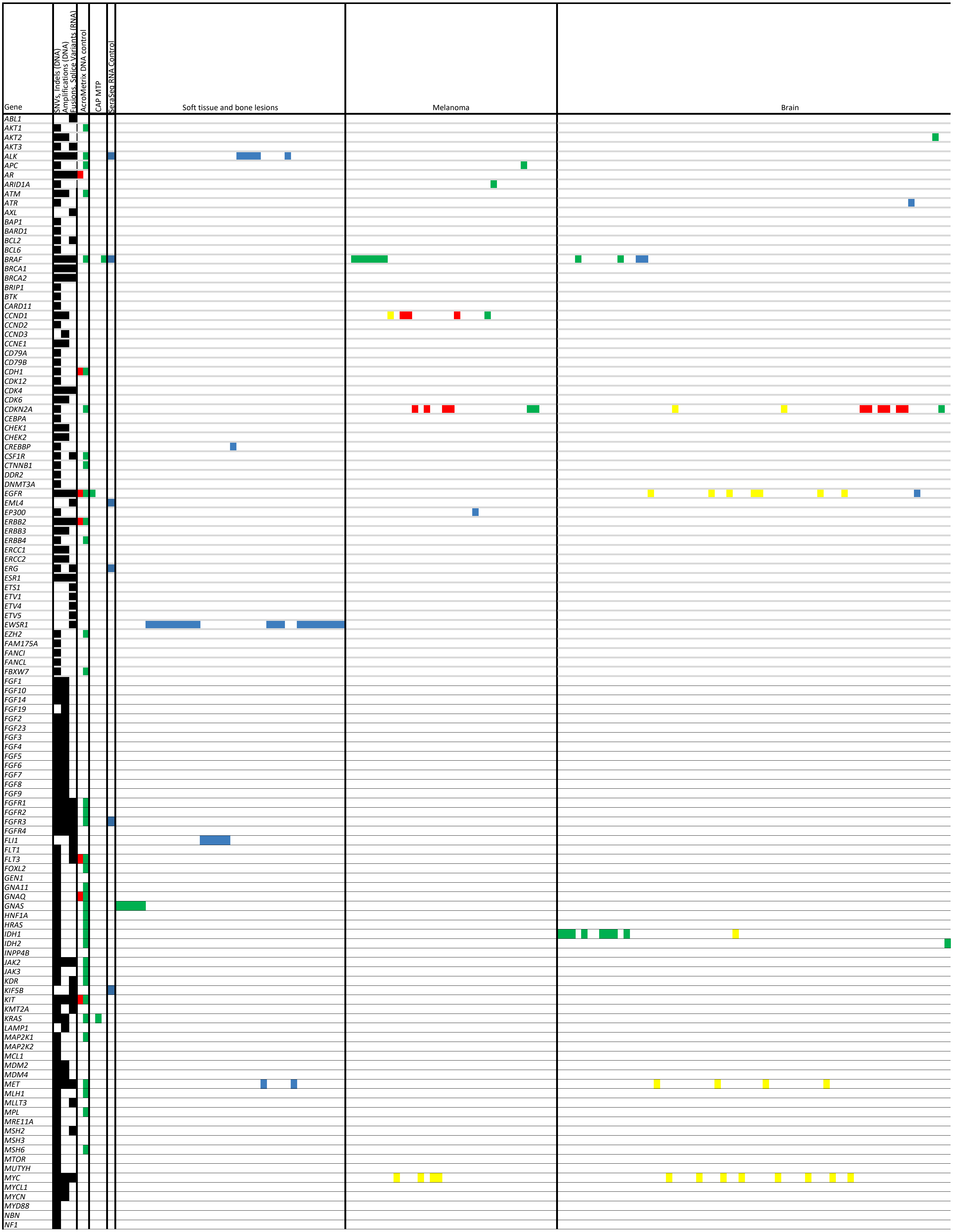
Gene
SNVs, Indels (DNA)
Amplifications (DNA)
Fusions, Splice Variants (RNA)
AcroMetrix DNA control
CAP MTP
SeraSeq RNA Control
Soft tissue and bone lesions
Melanoma
Brain
ABL1
AKT1
AKT2
AKT3
ALK
APC
AR
ARID1A
ATM
ATR
AXL
BAP1
BARD1
BCL2
BCL6
BRAF
BRCA1
BRCA2
BRIP1
BTK
CARD11
CCND1
CCND2
CCND3
CCNE1
CD79A
CD79B
CDH1
CDK12
CDK4
CDK6
CDKN2A
CEBPA
CHEK1
CHEK2
CREBBP
CSF1R
CTNNB1
DDR2
DNMT3A
EGFR
EML4
EP300
ERBB2
ERBB3
ERBB4
ERCC1
ERCC2
ERG
ESR1
ETS1
ETV1
ETV4
ETV5
EWSR1
EZH2
FAM175A
FANCI
FANCL
FBXW7
FGF1
FGF10
FGF14
FGF19
FGF2
FGF23
FGF3
FGF4
FGF5
FGF6
FGF7
FGF8
FGF9
FGFR1
FGFR2
FGFR3
FGFR4
FLI1
FLT1
FLT3
FOXL2
GEN1
GNA11
GNAQ
GNAS
HNF1A
HRAS
IDH1
IDH2
INPP4B
JAK2
JAK3
KDR
KIF5B
KIT
KMT2A
KRAS
LAMP1
MAP2K1
MAP2K2
MCL1
MDM2
MDM4
MET
MLH1
MLLT3
MPL
MRE11A
MSH2
MSH3
MSH6
MTOR
MUTYH
MYC
MYCL1
MYCN
MYD88
NBN
NF1

Gene	SNVs, Indels (DNA)	Amplifications (DNA)	Fusions, Splice Variants (RNA)	AcroMetrix DNA control	CAP MTP	SeraSeq RNA Control	Soft tissue and bone lesions	Melanoma	Brain
NOTCH1									
NOTCH2									
NOTCH3									
NPM1									
NRAS									
NRG1									
NTRK1									
NTRK2									
NTRK3									
PALB2									
PAX3									
PAX7									
PDGFRA									
PDGFRB									
PIK3CA									
PIK3CB									
PIK3CD									
PIK3CG									
PIK3R1									
PMS2									
PPARG									
PPP2R2A									
PTCH1									
PTEN									
PTPN11									
RAD51									
RAD51B									
RAD51C									
RAD51D									
RAD54L									
RAF1									
RB1									
RET									
RICTOR									
ROS1									
RPS6KB1									
SLX4									
SMAD4									
SMARCB1									
SMO									
SRC									
STK11									
TERT									
TET2									
TFRC									
TMPRSS2									
TP53									
TSC1									
TSC2									
VHL									
XRCC2									

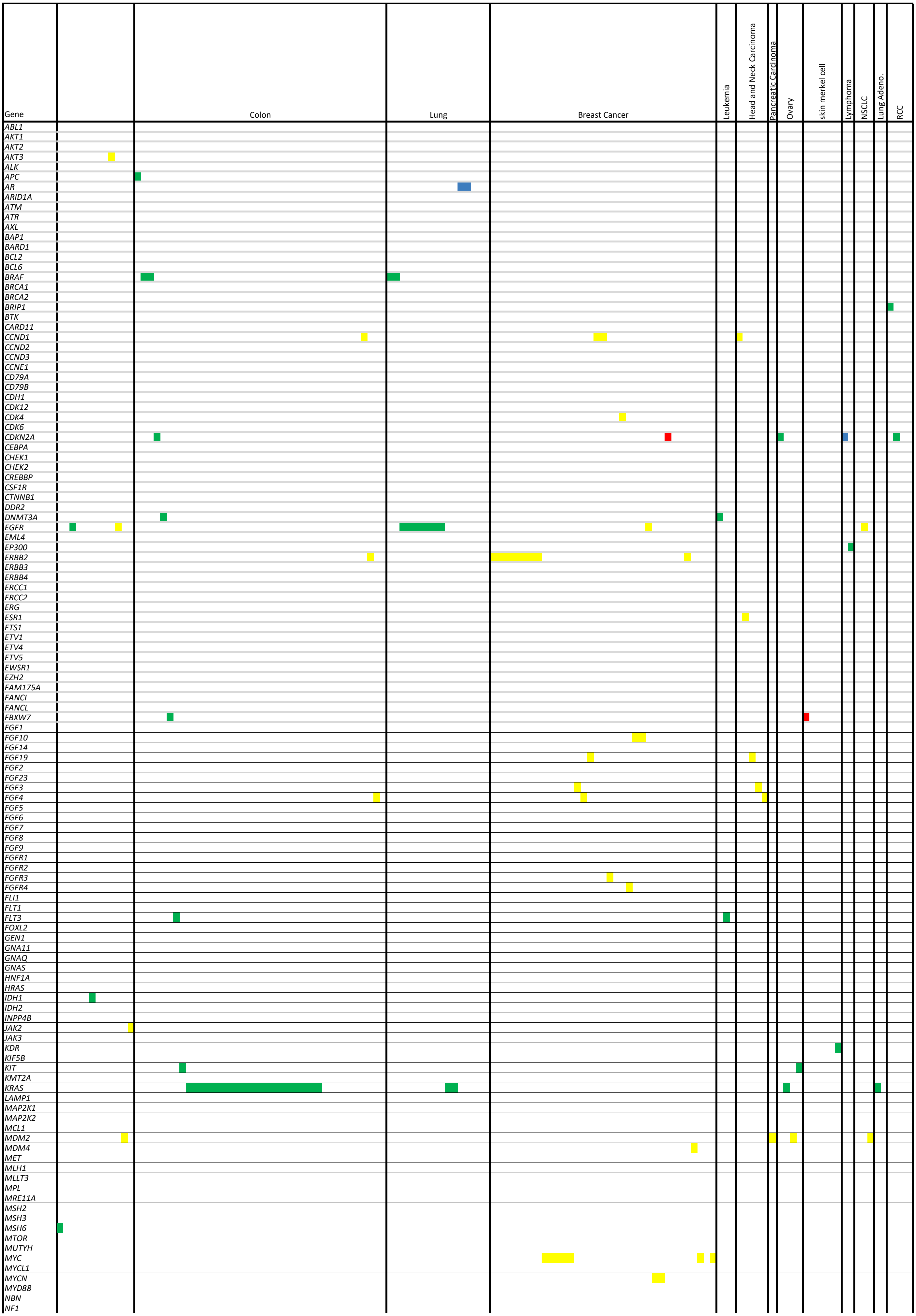
Gene
Colon
Lung
Breast Cancer
Leukemia
Head and Neck Carcinoma
Pancreatic Carcinoma
Ovary
skin merkel cell
Lymphoma
NSCLC
Lung Adeno.
RCC
ABL1
AKT1
AKT2
AKT3
ALK
APC
AR
ARID1A
ATM
ATR
AXL
BAP1
BARD1
BCL2
BCL6
BRAF
BRCA1
BRCA2
BRIP1
BTK
CARD11
CCND1
CCND2
CCND3
CCNE1
CD79A
CD79B
CDH1
CDK12
CDK4
CDK6
CDKN2A
CEBPA
CHEK1
CHEK2
CREBBP
CSF1R
CTNNB1
DDR2
DNMT3A
EGFR
EML4
EP300
ERBB2
ERBB3
ERBB4
ERCC1
ERCC2
ERG
ESR1
ETS1
ETV1
ETV4
ETV5
EWSR1
EZH2
FAM175A
FANCI
FANCL
FBXW7
FGF1
FGF10
FGF14
FGF19
FGF2
FGF23
FGF3
FGF4
FGF5
FGF6
FGF7
FGF8
FGF9
FGFR1
FGFR2
FGFR3
FGFR4
FLI1
FLT1
FLT3
FOXL2
GEN1
GNA11
GNAQ
GNAS
HNF1A
HRAS
IDH1
IDH2
INPP4B
JAK2
JAK3
KDR
KIF5B
KIT
KMT2A
KRAS
LAMP1
MAP2K1
MAP2K2
MCL1
MDM2
MDM4
MET
MLH1
MLLT3
MPL
MRE11A
MSH2
MSH3
MSH6
MTOR
MUTYH
MYC
MYCL1
MYCN
MYD88
NBN
NF1

Gene		Colon	Lung	Breast Cancer	Leukemia	Head and Neck Carcinoma	Pancreatic Carcinoma	Ovary	skin merkel cell	Lymphoma	NSCLC	Lung Adeno.	RCC
NOTCH1													
NOTCH2													
NOTCH3													
NPM1													
NRAS													
NRG1													
NTRK1													
NTRK2													
NTRK3													
PALB2													
PAX3													
PAX7													
PDGFRA													
PDGFRB													
PIK3CA													
PIK3CB													
PIK3CD													
PIK3CG													
PIK3R1													
PMS2													
PPARG													
PPP2R2A													
PTCH1													
PTEN													
PTPN11													
RAD51													
RAD51B													
RAD51C													
RAD51D													
RAD54L													
RAF1													
RB1													
RET													
RICTOR													
ROS1													
RPS6KB1													
SLX4													
SMAD4													
SMARCB1													
SMO													
SRC													
STK11													
TERT													
TET2													
TFRC													
TMPRSS2													
TP53													
TSC1													
TSC2													
VHL													
XRCC2													
